# Microbiota-Derived Regulation of AhR and VDR Signaling in Intestinal Inflammation: Protective Roles of Prebiotics, Probiotics, and Postbiotics

**DOI:** 10.3390/ijms27031295

**Published:** 2026-01-28

**Authors:** Fu-Chen Huang

**Affiliations:** Department of Pediatrics, Kaohsiung Chang Gung Memorial Hospital, Chang Gung University College of Medicine, Kaohsiung 833401, Taiwan; huang817@cgmh.org.tw; Tel.: +886-7-7317123 (ext. 8724)

**Keywords:** *Salmonella*, colitis, prebiotics, probiotics, postbiotics, AhR, VDR, SCFA, IL-22, tight junctions

## Abstract

Microbiota-derived indoles and short-chain fatty acids (SCFAs) modulate intestinal immunity via the aryl hydrocarbon receptor (AhR) and vitamin D receptor (VDR). This review proposes an operational AhR–VDR axis—three testable models (sequential, parallel, reciprocal)—to explain how indoles (AhR) and SCFAs/vitamin D (VDR) may cooperate to drive IL-22–mediated repair, antimicrobial peptide production, autophagy, and tight-junction restoration. We critically evaluate prebiotics, probiotics, and postbiotics: prebiotics shift fermentation toward SCFAs but show context-dependent effects; probiotics can supply indole-type AhR ligands yet are strain-specific; postbiotics offer standardized ligand delivery but face formulation challenges. We distinguish *Salmonella*-specific findings (e.g., SCFA suppression of SPI-1) from general colitis data and prioritize molecular validation, temporal mapping, multi-omics responder stratification, and standardized postbiotic development for clinical translation.

## 1. Introduction

*Salmonella enterica* serovars remain among the most common food-borne pathogens, responsible for significant global morbidity and mortality [1]. Following ingestion, *Salmonella* invades intestinal epithelial cells, eliciting robust inflammation and disrupting epithelial tight junctions (TJs) [2,3]. The intestinal microbiota acts as a primary line of defense through colonization resistance, nutrient competition, and metabolic end-products such as short-chain fatty acids (SCFAs) [4,5]. Dysbiosis enhances *Salmonella* adherence and invasion, leading to mucosal damage and inflammation [6].

Microbiota-targeted interventions—including prebiotics, probiotics, and postbiotics—offer potential for restoring host–microbe balance and reducing intestinal injury [7,8]. Prebiotics are fermentable dietary substrates that selectively stimulate beneficial bacteria, leading to production of SCFAs [9,10]. Probiotics are live microorganisms that confer health benefits via modulation of immune and epithelial functions [8]. Postbiotics, defined as non-viable microbial products or metabolites conferring physiological benefit, offer standardization advantages [7].

Our group has elucidated that microbial metabolites, including indole-3-carbinol (I3C) and butyrate, act via aryl hydrocarbon receptor (AhR) and vitamin D receptor (VDR) to regulate autophagy, antimicrobial peptides (AMPs), and epithelial renewal [11,12,13,14]. Activation of these nuclear receptors up-regulates IL-22 [15] and LL-37 and suppresses NF-κB signaling, collectively mitigating *Salmonella*-driven inflammation. AhR activation by indole derivatives induces IL-22 and antimicrobial peptide production [15,16,17], while VDR activation promotes autophagy and TJ restoration [12,18,19]. Understanding the interplay between microbial metabolism and host receptor signaling is critical for designing microbiota-based therapeutics against *Salmonella* colitis. This review integrates current mechanistic and translational findings on how prebiotics, probiotics, and postbiotics exert protective effects against *Salmonella* colitis.

## 2. Salmonella-Specific vs. General Inflammatory Mechanisms

Following ingestion of contaminated food or water, *Salmonella enterica* crosses the intestinal epithelium via M cells and enterocytes using its type III secretion system (T3SS) effectors such as SopE, SipA, and SipB [1]. These effectors manipulate the host cytoskeleton, facilitating invasion and activating pro-inflammatory cascades. Once intracellular, *Salmonella* engages host pattern-recognition receptors (PRRs), particularly Toll-like receptors (TLRs) [20] and NOD-like [21,22] receptors (NLRs), triggering the production of pro-inflammatory cytokines such as IL-1β, IL-6, and TNF-α [23,24,25]. Infected epithelial cells release chemokines, including CXCL1 and CXCL2, which recruit neutrophils through CXCR2-mediated signaling [26,27]. Although this inflammatory response contributes to bacterial clearance, it simultaneously generates reactive oxygen and nitrogen species—most notably oxygen and nitrate—that *Salmonella* exploits as alternative electron acceptors for aerobic respiration [26]. The ensuing neutrophil influx and epithelial apoptosis further compromise mucosal integrity. The oxidative environment thus created favors *Salmonella* survival and proliferation, enabling it to outcompete obligate anaerobes and persist within the inflamed gut [2,28].

Tight-junction integrity, mediated by proteins such as occludin, claudins, and ZO-1, is commonly disrupted during enteric infection [3,29,30], increasing epithelial permeability and enabling bacterial translocation. In *Salmonella* models, this TJ disruption is driven both by direct bacterial effectors (e.g., T3SS) and by host inflammatory mediators (neutrophil proteases, ROS). Microbiota-derived metabolites and host nuclear receptors can counteract these effects: AhR activation by microbial tryptophan metabolites (e.g., indole derivatives) promotes IL-22 and antimicrobial peptide (AMP) expression [15,16,31], supporting epithelial regeneration and mucosal defense. VDR signaling has been linked to autophagy induction and regulation of TJ-related gene expression [32,33,34], which can limit intracellular bacterial survival and support barrier restoration. Evidence for additive or synergistic effects of combined AhR and VDR activation exists in several preclinical reports, but most data are functional and inferential rather than demonstrating direct receptor–receptor interaction.

## 3. Effects of Prebiotics

Prebiotics—non-digestible substrates selectively utilized by beneficial gut bacteria—have profound effects on intestinal homeostasis [35]. Prebiotics (FOS, GOS, inulin, resistant starches, and human milk oligosaccharides) primarily act by enriching SCFA-producing taxa and increasing luminal butyrate and propionate (Table 1) [8,36]. SCFAs support colonocyte energy metabolism, enhance tight-junction protein expression (occludin, claudins, ZO-1), and modulate immune signaling via GPR41/43/109A and HDAC inhibition [9,37,38]. In *Salmonella* models, resistant starch and rice-bran supplementation have been associated with increased butyrate producers and reduced pathogen shedding or invasion [39]. HMOs such as 2′-FL improve barrier function and reduce pathogen adhesion in infant and colitis models [40,41,42,43,44,45,46,47,48,49,50,51,52]. providing mechanistic rationale for their protective potential in enteric infections.

Critical appraisal: Evidence is moderate. Mechanistic links (SCFA → AMP/AMPK/TJ) are well supported in vitro and in rodents, but human data are limited and variable. Outcomes depend on baseline microbiota composition, diet, and dose; some studies report context-dependent adverse effects (e.g., certain inulin formulations under specific dietary conditions). Future studies should include metabolomic endpoints, dose–response assessments, and stratification by baseline microbiota to identify responder phenotypes.

Axis link: Prebiotics feed the AhR–VDR axis primarily by increasing SCFAs that can enhance VDR expression/function and support autophagy and TJ restoration [32,33,34].

## 4. Effects of Probiotics

Probiotics, defined as live microorganisms that confer health benefits when administered in adequate amounts, modulate barrier and immune functions [7,8]. Probiotics can act through multiple mechanisms: competitive exclusion [63], enhancement of mucin and TJ proteins [53,55,64,65], and production of bioactive metabolites (SCFAs, indole derivatives) [55,56,62,66,67] (Table 2). Certain *Lactobacillus* and *Bifidobacterium* strains metabolize tryptophan into indole derivatives (e.g., indole-3-lactic acid) that act as AhR agonists and stimulate IL-22 and antimicrobial peptide production in preclinical systems [59,60,61]. In animal *Salmonella* models, pre-colonization with selected lactobacilli reduces colonization and pathology [68,69] supporting a potential protective role.

Critical appraisal: Evidence is heterogeneous. While mechanistic in vitro and animal data are robust for some strains, human randomized trials show inconsistent efficacy. Probiotic benefits are often strain-specific and transient; reproducible clinical benefit requires standardized strain selection, viability assurance, and demonstration of in vivo metabolite production at mucosal sites.

Axis link: Probiotics contribute to the axis mainly by producing indole-type AhR ligands and by indirectly promoting SCFA producers; strain selection should be guided by demonstrated indole production and in vivo activity.

## 5. Effects of Postbiotics

Postbiotics—non-viable microbial cells, components, or metabolites conferring health benefits—provide standardized, safe, and reproducible interventions [7]. Postbiotics (SCFAs, defined indole derivatives, bacteriocins, exopolysaccharides) provide direct receptor engagement without reliance on live microbes [7,11,74]. SCFAs (butyrate, propionate) modulate epithelial metabolism [75,76], GPR signaling [77,78], and HDAC activity [79,80]; indole derivatives activate AhR and induce IL-22/AMPs in preclinical models [16,31,81] (Table 3).

Critical appraisal: Postbiotic evidence is promising but largely preclinical. Key translational challenges include optimal composition, mucosal delivery, pharmacokinetics, and whether postbiotics can reproduce the adaptive signaling complexity of live microbes. Standardized formulation and PK studies, along with early-phase human trials including mechanistic biomarkers, are priorities.

Axis link: Postbiotics provide direct ligand delivery to engage AhR and VDR pathways, offering a translational route to test axis hypotheses in humans with controlled dosing.

## 6. Integration and Critical Synthesis

The intestinal barrier integrates microbial, metabolic, and immune signals to maintain mucosal equilibrium [3]. Prebiotics, probiotics, and postbiotics converge on overlapping molecular routes but differ in entry points, kinetics, and reproducibility. Below we synthesize these differences and critically evaluate evidence quality and contradictions.

### 6.1. SCFA–AMPK–Tight-Junction Axis: Evidence and Limits

SCFAs (butyrate, propionate, acetate) produced by fermentation of prebiotics activate metabolic regulators (AMPK, PPARγ), enhance mitochondrial function, and support TJ protein expression in multiple models [9,79,86]. SCFAs also signal via GPR41/43/109A to modulate immune responses (IL-10 induction, NF-κB suppression) [87]. However, much of the mechanistic detail derives from in vitro epithelial cell studies and murine colitis models; human data are more limited and confounded by diet and microbiota variability. Notably, some studies report context-dependent or adverse outcomes (e.g., certain inulin formulations worsening pathogen outcomes under specific diets), indicating that SCFA increases are not universally protective and that fermentation profiles matter.

### 6.2. AhR–IL-22 and VDR–Autophagy Crosstalk: Evidence and Caveats

AhR activation by indole derivatives robustly induces IL-22 in ILC3s and Th17 cells in multiple preclinical systems, promoting AMP expression [16,31,61,85,88,89] and epithelial repair [90,91]. IL-22 subsequently stimulates AMPs (RegIIIγ, LL-37) and epithelial regeneration. VDR signaling is associated with autophagy induction and antimicrobial peptide expression in epithelial cells, and vitamin D supplementation modulates these pathways in some models [14,33,92]. Reports of additive effects from combined AhR + VDR activation exist [14,84], but these are primarily from animal or ex vivo studies and often infer receptor interaction from downstream readouts rather than direct receptor–receptor assays. Therefore, while the hypothesis of functional synergy is plausible, it should be presented as provisional pending molecular validation (e.g., ChIP-seq, co-IP).

### 6.3. Prebiotics: Predictable Mechanism, Unpredictable Outcomes

Prebiotics reliably increase SCFA production and thereby engage VDR-linked pathways that enhance TJ proteins and epithelial metabolism; however, host microbiota composition and diet alter fermentation outcomes, producing context-dependent effects. Notably, some studies report that rapidly fermentable FOS/inulin decreased resistance to *Salmonella* in rodents unless buffering nutrients (e.g., calcium) were provided [93], illustrating that prebiotics can be protective or deleterious depending on host context, and underscoring the need for stratified, dose–response studies and metabolomic endpoints before broad recommendations [83].

### 6.4. Probiotics: Strain-Level Metabolite Production and Reproducibility Limits

Certain probiotic strains produce indole derivatives (indole-3-lactic acid, indole-3-aldehyde) that act as AhR agonists and stimulate IL-22 [94]; in vitro screens and engineered strains confirm strain-specific indole output and downstream immunomodulation [95]. The translational challenge is reproducibility: colonization is often transient and metabolic output varies with substrate availability and host factors.

### 6.5. Postbiotics: Translational Advantages and Unresolved Formulation Science

Postbiotics (standardized SCFAs, defined indoles, bacteriocins) meet ISAPP criteria for reproducible, non-viable interventions and offer safety and dosing advantages for clinical translation [7]. Yet whether postbiotics can fully recapitulate the dynamic, context-sensitive signaling of live microbes remains uncertain; formulation, mucosal delivery, and pharmacokinetics require systematic study.

### 6.6. Translational Gaps, Biomarkers, and Safety Considerations (Table 4)

Key gaps include: (1) molecular validation of AhR–VDR crosstalk (ChIP-seq, co-IP); (2) responder stratification using multi-omics to predict who benefits from prebiotic/probiotic/postbiotic regimens; (3) standardization of strains, doses, and postbiotic formulations; and (4) long-term safety of chronic receptor modulation (AhR xenobiotic pathways; VDR/calcium homeostasis). These priorities will enable precision interventions and reduce contradictory outcomes across studies.

**Table 4:** Comparative Effects of Prebiotics, Probiotics, and Postbiotics on AhR–VDR Signaling and *Salmonella* outcomes.

Intervention	Primary Target	Timing	Advantage	Main Caveat
Prebiotics	SCFA → VDR (indirect)	Slow	Durable microbiota reshaping	Host-dependent fermentation
Probiotics	Indole → AhR (strain)	Rapid, transient	Direct metabolite supply	Strain specificity
Postbiotics	Direct ligands	Immediate (dose)	Standardized dosing; safety	Delivery/formulation challenges

→: lead to.

## 7. Translational Perspectives

### 7.1. Clinical and Preclinical Evidence

Randomized trials demonstrate probiotic (*Lactobacillus rhamnosus*, *Bifidobacterium breve*) supplementation reduces infectious diarrhea duration and *Salmonella* shedding [96,97]. Synbiotics combining FOS or GOS with probiotics further enhance mucosal IgA and IL-22 responses [98,99]. Postbiotic preparations—heat-killed *Lactobacillus* cells or metabolite filtrates—show comparable anti-inflammatory benefits without viability concerns [7].

### 7.2. Precision Nutrition and Metabolomics

High-resolution metabolomic profiling now enables precision tailoring of biotic therapies. Distinct microbial and metabolic fingerprints correlate with responsiveness to SCFA- or indole-based interventions [16]. Functional foods enriched with AhR/VDR ligands—including indoles, vitamin D_3_, and 2′-FL—may synergize with probiotics to promote durable mucosal protection [13]. In particular, the IL-22 axis plays a pivotal role in maintaining the intestinal microenvironment and represents a promising dietary target for mucosal repair and host defense modulation [91]. The integration of metabolomics and transcriptomics will refine dose selection, identify responsive host–microbe signatures, and facilitate biomarker discovery for the rational design of future *Salmonella*-targeted nutraceuticals [30,100,101].

### 7.3. Translational Priorities and Specific Research Questions

To translate mechanistic insights into clinical interventions, we propose the following priorities:

- Molecular validation: Determine whether AhR and VDR co-occupy promoters of key antimicrobial or TJ genes (ChIP-seq), identify shared co-regulators, and test for physical interactions (co-IP).
- Temporal mapping: Characterize the kinetics of AhR and VDR activation during Salmonella infection and after administration of indoles, SCFAs, or vitamin D.
- Responder stratification: Use metagenomics and metabolomics to identify microbiome/metabolome signatures that predict responsiveness to prebiotic/probiotic/postbiotic regimens.
- Formulation science: Develop postbiotic delivery systems (encapsulation, prodrugs) that achieve effective mucosal concentrations with minimal systemic exposure.
- Adaptive clinical trials: Design trials that stratify participants by baseline microbiome/metabolome profiles and include mechanistic endpoints (IL-22, LL-37, VDR expression, SCFA levels).

## 8. Critical Appraisal of Key and Emerging Evidence in Microbiota–Host Signaling

Several seminal studies have established the conceptual foundation linking microbiota-derived metabolites to epithelial protection and immune regulation. Classic work by Fukuda et al. demonstrated that acetate produced by *Bifidobacterium* enhances epithelial defense against enteropathogens through reinforcement of barrier integrity, highlighting the protective potential of SCFAs beyond their nutritional role [4]. Similarly, Peng et al. provided mechanistic evidence that butyrate strengthens TJ assembly via AMPK activation, a finding that has since been widely extrapolated to multiple colitis models [9]. While these studies remain foundational, they were largely conducted in simplified systems or non-*Salmonella* contexts, underscoring the need for cautious interpretation when extending conclusions to pathogen-driven colitis.

More recent investigations have expanded the scope of SCFA biology, demonstrating immunomodulatory effects mediated through GPCR signaling and epigenetic regulation [79,102]. However, conflicting findings have emerged regarding SCFA availability during acute *Salmonella* infection, as inflammatory oxygenation of the gut lumen may suppress obligate anaerobes responsible for butyrate production [2]. This paradox raises an important unresolved question: whether SCFA supplementation or postbiotic delivery can fully compensate for inflammation-induced metabolic disruption in vivo.

The identification of AhR as a central mediator of microbiota–host crosstalk represents a major conceptual advance. Zelante et al. and Lamas et al. elegantly demonstrated that microbial tryptophan metabolites activate AhR to induce IL-22–dependent mucosal protection [16,31]. These studies provided compelling mechanistic insights but relied primarily on DSS-induced colitis and genetic immune perturbation models. Subsequent work, including our own, has extended these observations to *Salmonella*-induced colitis, demonstrating that probiotic-derived indoles and dietary AhR ligands attenuate pathogen-induced inflammation and bacterial burden [84]. Nevertheless, the relative contribution of epithelial versus immune cell–specific AhR signaling remains incompletely resolved, representing a key gap for future investigation.

VDR signaling has similarly emerged as an essential, yet under-integrated, component of intestinal host defense. Early studies established that vitamin D deficiency exacerbates experimental colitis and impairs antimicrobial peptide production [11,103]. More recent work has revealed that VDR activity intersects with microbial signals, autophagy pathways, and TJ regulation [104,105]. Despite these advances, direct mechanistic links between specific microbiota-derived metabolites and VDR activation remain sparse, and many conclusions are inferred from correlative or supplementation studies. This represents a notable limitation in the current literature and highlights an opportunity for targeted mechanistic studies.

Importantly, only a limited number of studies have explored coordinated AhR–VDR signaling. While functional convergence on IL-22 induction, autophagy, and epithelial repair has been observed, definitive molecular crosstalk between these nuclear receptors has not been fully elucidated. Our previous findings [84,104] suggest additive or synergistic protection when both pathways are engaged during *Salmonella* infection, but whether this reflects shared transcriptional targets, epigenetic cooperation, or sequential signaling remains unresolved.

Recent advances in postbiotic research have further complicated this landscape. The ISAPP consensus has emphasized the translational appeal of postbiotics due to their stability and safety profile [7]. However, the field currently lacks consensus on optimal composition, dosing, and long-term consequences of sustained receptor activation. In particular, whether chronic exposure to high-affinity AhR ligands could have adverse immunological effects remains controversial and insufficiently studied.

Collectively, these findings highlight both the strength and the fragmentation of the existing literature. While substantial progress has been made in delineating individual pathways, integrative studies directly comparing prebiotics, probiotics, and postbiotics within the same experimental framework remain scarce. This fragmentation may partly explain inconsistent outcomes across models and clinical studies and underscores the need for systems-level approaches.

## 9. Limitations and Future Directions

Despite substantial mechanistic and preclinical evidence, the clinical application of microbiota-directed therapies for *Salmonella* colitis faces important challenges. Interindividual differences in host genetics, diet, and baseline microbiota composition determine therapeutic responsiveness [106]. Standardization of dose, strain selection, and intervention timing remains inconsistent among studies [107].

Prebiotic efficacy depends on microbiota capacity to ferment fibers into SCFAs, which varies significantly between individuals [108]. Likewise, probiotic persistence in the intestine is often transient and host-dependent [109]. Postbiotics, although safer, require reproducibility testing across manufacturing batches and host backgrounds [7].

Future studies should employ *multi-omics* (metagenomics, metabolomics, transcriptomics) to delineate microbial and host determinants of response [100,101]. Integration of these datasets with clinical endpoints—cytokine profiles, bacterial load, histopathology—will enable precise personalization of biotic interventions. Furthermore, combined strategies harnessing prebiotics, probiotics, and postbiotics may deliver additive benefits through complementary mechanisms of barrier repair, immune modulation, and microbial competition [12,13,85].

## 10. Conclusions

Microbiota-derived indoles and SCFAs converge on AhR and VDR to coordinate epithelial defense, but the AhR–VDR axis remains a working model that needs molecular validation and human mechanistic data. Future work should prioritize ChIP-seq/co-IP studies, temporal activation mapping, multi-omics stratification of responders, and standardized postbiotic formulation and PK trials to enable precision therapeutics.

## Figures and Tables

**Table 1:** Probiotic strains, immunoregulatory signaling pathways, and intestinal protective outcomes.

Probiotic Type/Key Species	Primary Protective Actions	Barrier Mechanisms (Tight Junctions/Mucus)	Immunomodulatory Pathways (PRRs + Cytokines)	Functional Outcomes in Intestinal Defense	Supporting References
Classic microbiota-directed probiotics (*Lactobacillus*, *Bifidobacterium*, *Enterococcus*, *Saccharomyces*)	Competitive exclusion; ↓ pathogen invasion	Maintain epithelial adherence junctions	TLR2/TLR4 balancing; ↓ IL-6, TNF-α; ↑ IL-10	↓ Mucosal inflammation; ↑ epithelial resilience	[7,8,13,53,54]
Tight-junction regulatory probiotics *Lactobacillus* spp., *Bifidobacterium* spp.	↓ Permeability; ↑ TJ repair	↑ Claudin-1, Occludin, ZO-1; ↑ MUC2/MUC3	PRR cross-talk limiting LPS-NF-κB activation	Improved sealing and wound healing	[55,56,57,58]
AhR-activating indole-converting probiotics	↑ AMP secretion; Enhanced immune–epithelial crosstalk	Supports epithelial renewal via IL-22	AhR → IL-22/IL-17C; ↑ RegIIIγ & LL-37	↑ Pathogen killing; ↑ crypt regeneration	[16,59,60,61]
VDR-augmenting probiotic & nutrient synergy *B. longum* + Vitamin D	Autophagy induction	VDR-mediated junctional reinforcement	↓ IL-1β, TNF-α; ↑ cathelicidin (LL-37)	↓ Intracellular *Salmonella*; ↓ cytokine storm	[11,12,13,14]
Postbiotic-signaling probiotic derivatives (e.g., EVs, peptides)	AhR-mediated defense without live cells	Maintains barrier signaling indirectly	Non-viable ligands → AhR immune activation	Safe for immunocompromised hosts; retains efficacy	[62]

↓: decrease; ↑: increase; →: lead to.

**Table 2:** Functional roles and mechanisms of key human milk oligosaccharides (HMOs) in intestinal epithelial defense.

HMO Category	Representative HMOs	Primary Actions	Mechanistic Pathways	Functional Outcomes	Supporting References
Fucosylated HMOs	2′-FL, 3′-FL	Prebiotic enrichment of *Bifidobacterium* spp.; Anti-adhesion	Soluble decoy receptor → blocks fimbriae-dependent attachment; Microbial metabolic gene regulation	↓ *P. aeruginosa*, *C. jejuni*, ETEC adherence; ↓ diarrheal burden	[40,41,42,43,50]
2′-FL—high-abundance immunomodulatory HMO	2′-FL (major ~30% of HMOs)	Barrier protection; Immune modulation	↑ Tight-junction proteins (claudin-5/8); ↓ IL-6, TNF-α, IL-8, iNOS; ↑ SCFAs	↓ DSS/IL-6–induced colitis severity; Improved colon length, wound healing, epithelial permeability	[46,47,48,49,50,51]
Structural-specific HMOs for epithelial maturation	LNnT, 3′-FL, 6′-SL	Promote epithelial differentiation & mucus barrier integrity	Upregulation of claudins; Goblet-cell lineage support; Stem-cell renewal	Enhanced glycocalyx; ↑ resistance to pathogenic challenge	[45,52,70,71,72]
Synbiotic HMO strategies	2′-FL + 6′-SL; 2′-FL + GOS	Cooperative microbiota & host signaling modulation	↑ SCFA output; ↓ TLR-driven cytokine cascades; Restoration of tight junctions	↓ IL-6/TNF-α; ↓ epithelial injury; Improved tissue repair in colitis	[51,73]

↓: decrease; ↑: increase; →: lead to.

**Table 3:** Postbiotic categories, molecular mechanisms, and protective outcomes in intestinal health.

Postbiotic Type	Representative Molecules	Primary Receptors/Molecular Targets	Signaling Pathways	Functional Outcomes	Supporting References
**SCFA-based postbiotics**	Butyrate, Propionate	GPR41/FFAR3, GPR43/FFAR2, GPR109A	GPCR → MAPK signaling; NF-κB suppression; HDAC inhibition; IL-10/Treg induction	↓ Inflammation; ↑ barrier integrity; ↓ Salmonella SPI-1 expression and epithelial invasion	[77,79,82,83]
**Indole-derived postbiotics**	I3C, Indole-3-aldehyde, microbial tryptophan metabolites	AhR; IL-22 axis; Autophagy proteins	AhR → IL-22/IL-17C → AMP secretion; Autophagy activation; Tight-junction regulation	↓ Colitis severity; ↑ autophagic clearance; ↑ antimicrobial defense; ↑ barrier restoration during Salmonella infection	[16,81,84,85]
**Emerging postbiotic agents**	Bacterial peptides, cell-wall fragments, exopolysaccharides; *L. plantarum* supernatants	Pattern-recognition receptors; Immune regulatory nodes	PRR signaling modulation; Mucosal renewal cues	↓ Salmonella invasion; Accelerated epithelial regeneration; Safe for immunocompromised populations	[7,8,11,74]

↓: decrease; ↑: increase; →: lead to.

## Data Availability

No new data were created or analyzed in this study. Data sharing is not applicable to this article.

## Abbreviations

The following abbreviations are used in this manuscript:

SCFAs	Short Chain Fatty Acids
TJs	Tight junctions
I3C	Indole-3-carbinol
AhR	Aryl hydrocarbon receptor
VDR	Vitamin D receptor
AMPs	Antimicrobial peptides
T3SS	Type III secretion system
PRRs	Pattern-recognition receptors
TLRs	Toll-like receptors
NLRs	NOD-like receptors
FOS	Fructo-oligosaccharides
GOS	Galacto-oligosaccharides
HMOs	Human milk oligosaccharides
2′-FL	2′-fucosyllactose
AMPK	AMP-activated protein kinase
PPARγ	Peroxisome proliferator-activated receptor gamma
LNnT	Lacto-N-neotetraose
6′-SL	6′-Sialyllactose
HDACi	Histone deacetylase (HDAC) inhibitor
